# A polyoxomolybdate-based hybrid nano capsule as an antineoplastic agent†

**DOI:** 10.1039/d3na00459g

**Published:** 2023-10-06

**Authors:** Arti Joshi, Sobhna Acharya, Neeta Devi, Ruby Gupta, Deepika Sharma, Monika Singh

**Affiliations:** a Institute of Nano Science and Technology Knowledge City, Sector-81 Mohali Punjab India monika@inst.ac.in

## Abstract

Polyoxometalates (POMs) are versatile anionic clusters which have attracted a lot of attention in biomedical investigations. To counteract the increasing resistance effect of cancer cells and the high toxicity of chemotherapeutic treatments, POM-based metallodrugs can be strategically synthesized by adjusting the stereochemical and physicochemical features of POMs. In the present report a polyoxomolybdate (POMo) based organic–inorganic hybrid solid (C_6_H_16_N)(C_6_H_15_N)_2_[Mo_8_O_26_]·3H_2_O, solid 1, has been synthesized and its antitumoral activities have been investigated against three cancer cell lines namely, A549 (Lung cancer), HepG2 (Liver cancer), and MCF-7 (Breast cancer) with IC50 values 56.2 μmol L^−1^, 57.3 μmol L^−1^, and 55.2 μmol L^−1^ respectively. The structural characterization revealed that solid 1 consists of an octa molybdate-type cluster connected by three triethylamine molecules *via* hydrogen bonding interactions. The electron microscopy analysis suggests the nanocapsule-like morphology of solid 1 in the size range of 50–70 nm. The UV-vis absorption spectra were used to assess the binding ability of synthesized POM-based solid 1 to calf thymus DNA (ctDNA), which further explained the binding interaction between POMo and ctDNA and the binding constant was calculated to be 2.246 × 10^3^ giving evidence of groove binding.

## Abstract 2

Anti-tumoral properties of bio(organic) functionalized polyoxomolybdate. The synthesized hybrid nanocapsules exhibited excellent biocompatibility and stability at physiological pH making them efficient candidates for biomedicinal application.

## Graphical abstract

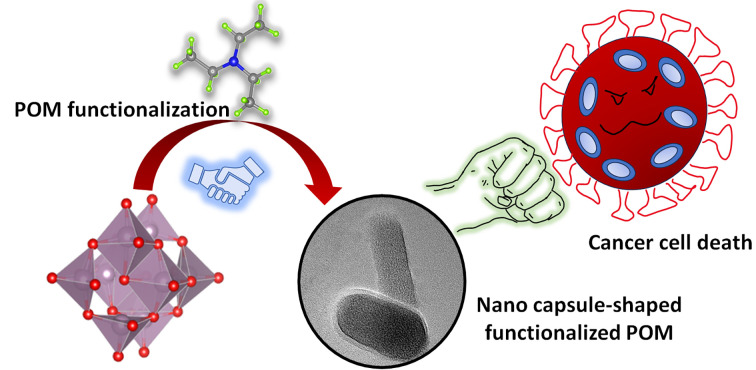

## Introduction

While the global pandemic COVID-19 has dramatically taken over millions of lives, another disease that still unpredictably continues to devastate us in silence is cancer. It is a disease so prevalent that neither can it be distinguished as an epidemic nor a pandemic.^1^ It has become a global threat and the need of the hour is to combat it. Cancer develops from prototype actuating agents in a group of cells causing DNA damage and, in turn leading to genetic mutations or altering the structure of chromosomes.^2,3^ These various modifications eventually result in permanent changes. DNA is a crucial component of each organism's genetic makeup and a main intracellular target for anticancer medications. Small molecule interactions with DNA can harm cancer cells and result in cell death.^4,5^

Despite cisplatin being one of the most commonly administered and widely used chemotherapeutic agents in clinics, a series of other drugs have also demonstrated their anticancer potential by temporarily alleviating symptoms, prolonging the lifespan of patients and in very rare circumstances, even curing cancer.^6–8^ However, their main drawback is the side effects resulting from a lack of selectivity, low bioavailability and less efficiency to some of the cancer types.^9,10^ Therefore, the quest for alternative novel, effective and non-toxic drugs is still ongoing. Polyoxometalates have come to the rescue as the next generation of potential metallodrugs to cure cancer.^11^

Polyoxometalates (POMs) are a distinct class of transition-metal oxide cluster anions that are typically constructed from group 5 and group 6 transition metals (V, Nb, Ta, Mo, and W)^12^ and have a wide range of structures in terms of size, shape, heteroatom, elemental composition and nuclearities.^13^ On account of this great diversity in structures and properties, POMs have been reported to have a varied range of applications in different fields starting from catalysis^14–20^ to electrochemistry,^13,21–23^ macromolecular crystallography^24^ biotechnology,^25^ nanotechnology,^26^ and in biochemistry and medicine.^25,27–31^ Some recent reports have been published which emphasized on POMs being used for biological imaging and as contrast agents for magnetic resonance imaging.^32^ POMs containing high nuclear charge (high-Z) elements act as effective radiosensitizers generating Compton and Auger electrons that interact with the surrounding water or oxygen molecules in order to produce ROS which in turn can be a boon to radiotherapy to cure hypoxic tumors.^33^

**Fig. 1:**
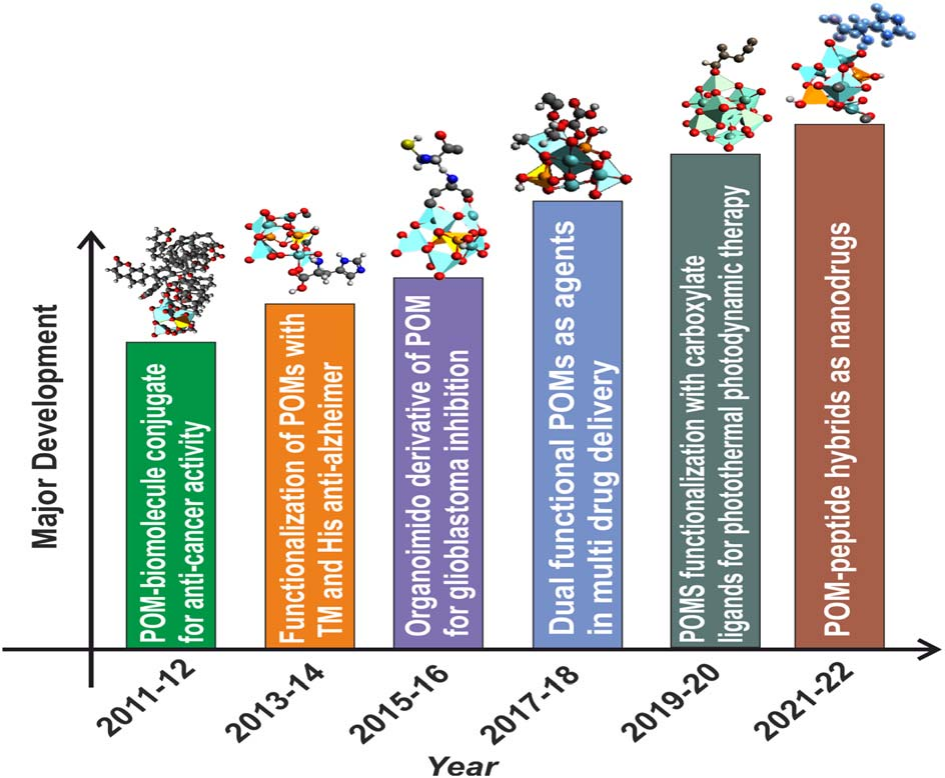
Major development in the biomedical applications of functionalized polyoxometalates with different classes of organic moieties. TM: transition metal; His: histidine.

The anticancer activity of POMs dates back to 1965, when Mukherjee vividly described the *in vivo* application of a mixture in patients suffering from gastrointestinal cancer.^34^ It was given the name PTMC, and was the combination of H_3_[PW_12_O_40_] (phosphotungstic acid), H_3_[PMo_12_O_40_] (phosphomolybdic acid) and caffeine. Although it resulted in the complete disappearance of tumors in four patients, PTMC was still not subjected to any additional clinical studies.^35^ A few years later, in 1974, Jasmin *et al.* attempted to describe the inhibitory effect of (NH_4_)_17_Na[NaSb_9_W_21_O_86_] against sarcoma virus-induced tumors which paved the path for the development of POMs which are biologically active.^36^ In this regard, [NH_3_Pr^i^]_6_[Mo_7_O_24_] (PM-8) was synthesized by Yamase and colleagues and has been tested for its anticancer properties *in vitro* and *in vivo* making some significant pioneering progress. Yamase also put up a single-electron theory to explain how POMs can prevent cancer.^36,37^ Despite the promising results that pure inorganic POMs have displayed against a variety of cancer types, their therapeutic use is hindered mainly due to their long-term cytotoxicity and low selectivity. Additionally, under physiological conditions, most POM-prototypes are thermodynamically and kinetically less stable.^11^ To overcome this limitation, research interest is now shifting from inorganic POMs to POM-based organic–inorganic hybrids^38^ because the functionalization or encapsulation of POMs with organic moieties not only boosted the POM's anticancer potential but also generally lowered the POM's toxicity.^12,39^ and improved the overall bioactivity of the system. Hybrid POMs can be differentiated into two major classes based on the type of interactions. Class-I hybrid POMs have an ionic interaction with various types of (bio) organic molecules, whereas Class-II hybrid POMs have a covalent link with their corresponding (bio)organic counterparts.^40^ Class II hybrids show improved stability and homogeneity. The functionalization strategies include encapsulation and grafting bioactive ligands into inorganic POM nuclei to minimize toxicity and provide synergistic effect of both clusters and ligands for enhancing biological targets.^41^ POM-biomolecule conjugates like tris(hydroxymethyl aminomethane), DHCA (dehydrocholic acid),^42^ imido derivatives,^43^ amino acids,^44^ peptides,^45^ and carboxylate based ligands^46^(*i.e.* folic acid, glycolic acid, *etc.*) have been reported to show anti-cancer, anti-Alzheimer's, and glioblastoma inhibition properties to name a few. Recently, a few reports have been published regarding the interaction of polyoxometalates with proteins, affecting various biological processes.^30,31,47^ The major advancement in this direction has been summarized in Fig. 1.^6,38,39,41,42,44–46^

The elemental questions regarding the mechanism behind POM-mediated antiproliferative activity are still up for debate. However, it is well accepted that POMs can show cancer cell penetration as their location has been experimentally confirmed within cytoplasmic space.^48,49^ One such mechanism was proposed by Yamase^37,50^ which suggested that due to repeated reduction/oxidation cycles between POM and cell components it interferes with ATP generation which finally leads to apoptosis. It is a very well-known fact that DNA damage is one of the main stimuli which induces apoptosis.^11,51^ So in some of the reports DNA interaction with POM was elucidated to be the mechanism of anticancer activity.^6,11,48,52,53^ Few more mechanisms of anti-tumor activity were found to be inhibition of angiogenesis and interaction of polyoxometalate with proteins.^4,6^

Herein, we have reported a β-octamolybdate type of POM cluster functionalized with triethyl amine molecules (C_6_H_16_N)(C_6_H_15_N)_2_[Mo_8_O_26_]·3H_2_O (1), and its structural composition was investigated by single crystal X-ray diffraction followed by other subsequent characterization techniques such as powder X-ray diffraction, FTIR, electron microscopy analysis (FESEM and TEM), X-ray photoelectron spectroscopy (XPS) and thermogravimetric analysis. It was found to show antitumor activities in three cancer cell lines namely lung cancer (A549), breast cancer (MCF-7), and liver cancer (HepG2) cells. The rationale behind opting for molybdenum based POM was due to its broad labile chemistry and low toxicity. Apart from being effective against all three cancer cells, it showed biocompatibility to normal cells. Also, it was found to have good stability in PBS buffer at physiological pH, overcoming the inherent drawback of pure POMs and further indicating it as a potential antitumor candidate. Further, an attempt has been made to elucidate the mechanism of the antiproliferation activity of the octamolybdate cluster-based inorganic–organic hybrid solid through DNA binding using UV-visible spectroscopy. Additionally, DNA damage through a DNA fragment assay was checked in order to elaborate the mechanism for cell death.

## Experimental

### Materials and methods

All the chemicals used were purchased from commercial sources and used as such without any further purification. The reagent, ammonium molybdate tetrahydrate, was obtained from CDH, and triethylamine was purchased from TCI.

### Synthetic procedure

A mixture of (NH_4_)_6_·Mo_7_O_24_·4H_2_O (1 mmol) and triethylamine (1 mmol) along with DMF (15 ml) and H_2_O (20 ml) was kept under stirring until a clear solution was obtained. It was then transferred into a 23 ml Teflon tube and heated solvothermally at 140 °C for 48 hours. A white coloured crystalline product was obtained. It was then filtered, and further washed with distilled water to remove unwanted products and then dried in air. The solvent used for studies was PBS (pH = 7.4) in which the white coloured crystalline product is completely soluble and becomes colourless. The stock solution (100 μM) was prepared once and then stored at 4 °C until use. Working solutions were made fresh according to the experiments.

### Cell culture studies

The human breast (MCF-7), lung (A549), and liver (HepG2) cancer cell lines and a healthy murine fibroblasts cell line (L929) were acquired from India's National Cell Repository NCCS, Pune and were kept in 10% fetal bovine serum (FBS; Gibco) in DMEM (Hyclone) at 37 °C, under 5% CO_2_ and 1% penicillin-streptomycin solution (Lonza).

### *In Vitro* biocompatibility and cytotoxicity assay

The A549, MCF-7, and HepG2 cancer cell lines were used to evaluate the cytotoxicity while the L929 cell line was used to measure the biocompatibility using the conventional MTT [3-(4, 5-dimetheylthiazol-2)-2,5-diphenyl tetrazolium bromide] assay. The detailed experimental technique for the cytotoxicity assay is provided in the ESI.†

### Other analytical techniques

Details of the instrumentation for powder X-ray diffraction (PXRD), FT-IR analysis, thermogravimetric analysis (TGA), X-ray Photoelectron Spectrometer (XPS) analysis are given in the ESI.†

### UV-visible spectroscopy

The UV-visible spectra were recorded on a UV 2600, Shimadzu using a 1 cm × 1 cm quartz cuvette for investigating the biocompatibility of the synthesized material and the interaction of the synthesized material with cellular DNA. Calf thymus DNA (ctDNA) was commercially purchased. The UV-visible spectra of POMo and the combination of POMo and ct-DNA were recorded in the wavelength range of 200–350 nm. All studies were performed in PBS buffer at pH 7.4 by gradually varying the ctDNA concentration.

## Results and discussion

### Structural elucidation of (C_6_H_16_N)(C_6_H_15_N)_2_[Mo_8_O_26_]·3H_2_O, 1

**Fig. 2:**
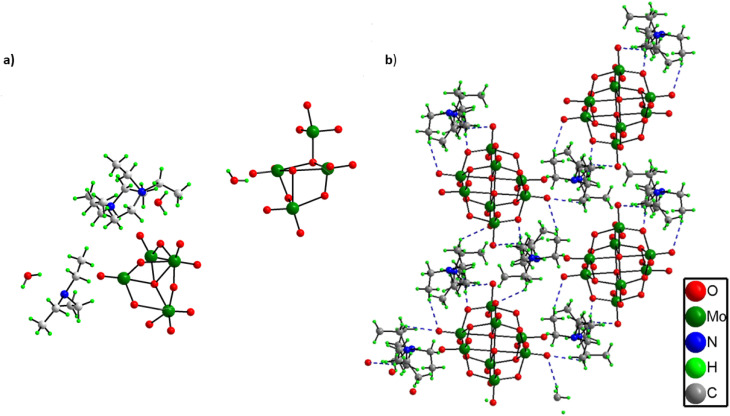
(a) Asymmetric unit and (b) Hydrogen bonded structure of (C_6_H_16_N)(C_6_H_15_N)_2_[Mo_8_O_26_]·3H_2_O (1).

From the single crystal X-ray diffraction it was found out that the asymmetric unit of solid 1 comprises two half octamolybdate cluster units, three triethylamine molecules and three lattice water molecules (Fig. 2a). All three triethylamines are protonated as confirmed by XPS analysis as well. Further the terminal oxygens of octamolybdate clusters show hydrogen bonding interactions with triethylamine molecules (C–H⋯O) and water molecules which leads to an extended framework structure of solid 1 (Fig. 2b). A crystallographic table has been added in the ESI.† (Table S1).

### Other analytical characterization

**Fig. 3:**
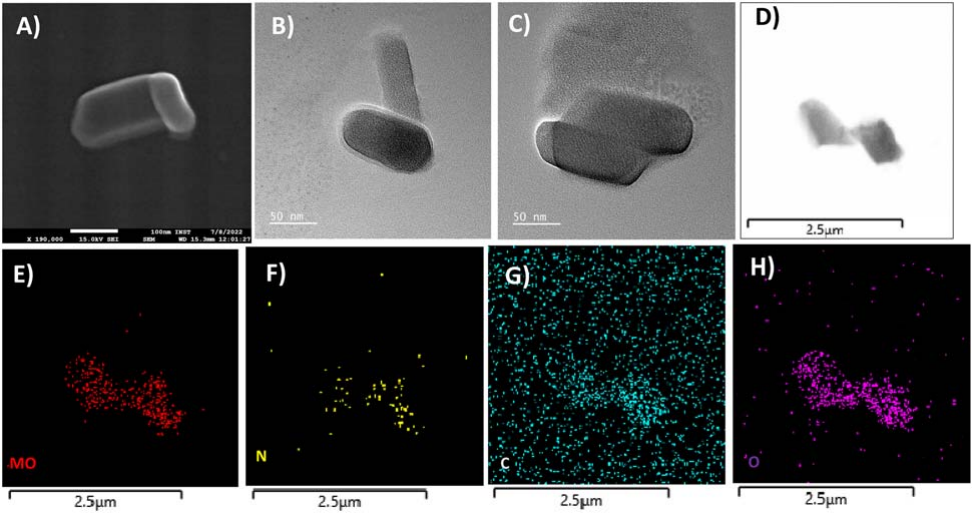
(A) FESEM image and (B-D) TEM images of solid 1 showing a nano-capsule shaped structure. (E–H) STEM mapping confirms the presence of all the elements detected by single-crystal X-ray diffraction and XPS analysis of solid 1.

The powder X-ray diffraction pattern for as-synthesized solid 1 is in good agreement with that of the simulated pattern which is indicative of phase purity of the synthesized material as shown in Fig. S1.† The thermogravimetric analysis (Fig. S2†) was performed from room temperature (25 °C) to 900 °C under an N_2_ atmosphere revealing that the thermal decomposition proceeded *via* three different steps. The first weight loss occurred between 0 and 58 °C attributed to the removal of water molecules (cal. 2.4% and exp 2.3%). The second weight loss appearing between 66 and 500 °C corresponds to the disruption of the ligand (triethylamine) (cal 20.2%, exp 22.3%). And at last, the third weight loss occurs above 500 signifying the breakdown of the octamolybdate cluster.^54,55^ The final residue is obtained at 890 °C which contributes to 78% of the initial mass. The FTIR spectrum shown in Fig. S3† exhibits strong peaks at 940 cm^−1^ which are ascribed to Mo–O stretching vibrations, while the bands appearing around 860 cm^−1^ are evident vibrations of Mo–O–Mo.^56^ The peaks at 933, 890, 845, and 651 cm^−1^ are attributed to the *ν*(Mo–O_t_) and *ν*(Mo–O_b_) vibrational modes of the [Mo_8_O_28_]^8−^ anion and the peaks between 400 and 860 specifically 849, 710, 666, and 551 cm^−1^ are due to β-octamolybdate.^55^ Additionally the appearance of the FTIR peak at 1420 cm^−1^ corresponds to the bending vibrations of the C–H bond supporting the existence of the ligand triethylamine.^57^ FESEM and TEM analyses revealed the nano-capsule-like morphology of the synthesized material in the size range of 50–200 nm (Fig. 3A–D, S5 and S6†). STEM mapping validates the presence of all the elements in solid 1 (Fig. 3E–H) which have been found from single crystal X-ray diffraction analysis. Additional electron microscopy images (FESEM and TEM) are shown in Fig. S5 and S6.†

**Fig. 4:**
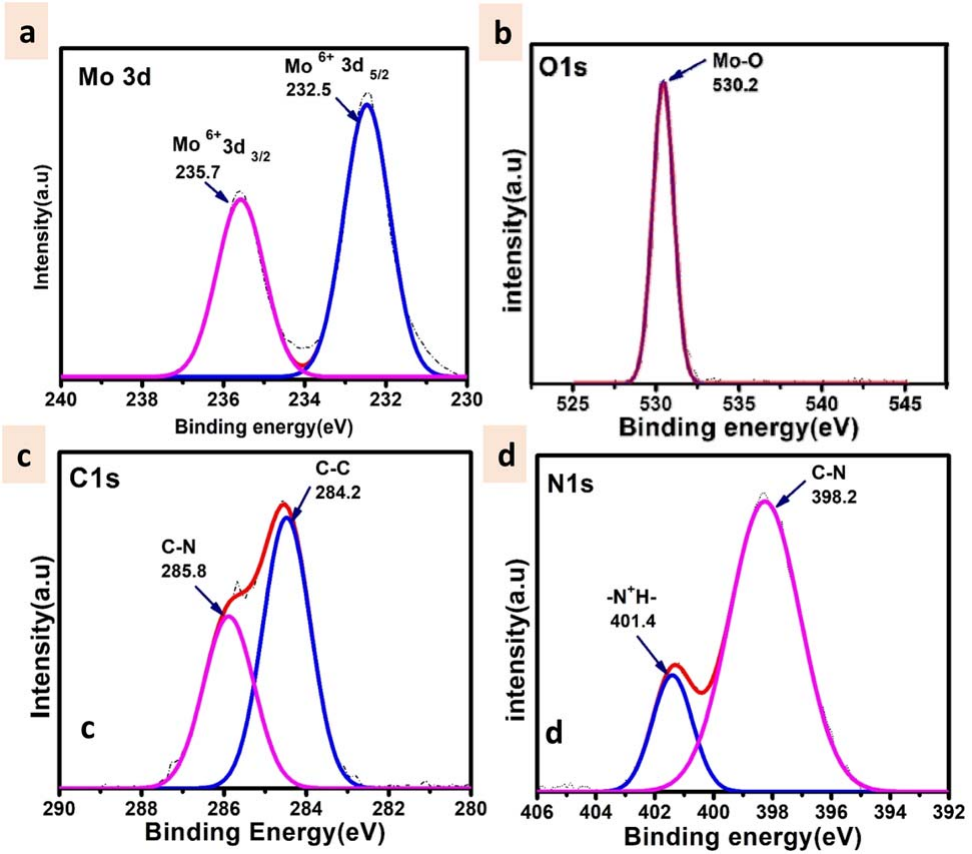
High resolution XPS spectra of (a) Mo 3d (b) O 1s (c) C 1s and (d) N 1s.

The XPS survey spectrum (shown in Fig. S4†) displays the existence of Mo, C, N, and O elements in the synthesized material. The Mo 3d_5/2_ and Mo 3d_3/2_ binding energy values were observed at 232.5 and 235.7 eV respectively (Fig. 4a) that can be evidence of the fact that molybdenum is present at its highest oxidation state *i.e.*, Mo^6+^. The fitted O 1s spectra show a single peak at 530.2 eV which corresponds to the Mo–O bond (Fig. 4b). The C 1s deconvoluted spectra display two peaks attributed to the C–C and C–N of the ligand at 284.2 and 285.8 respectively (Fig. 4c).^58^ Further, the N 1s spectra apparently show the protonated N peak at 401.4 eV supporting that the N of the triethylamine ligand might be present in protonated form in the cluster (Fig. 4d).^59^

### Stability measurements

**Fig. 5:**
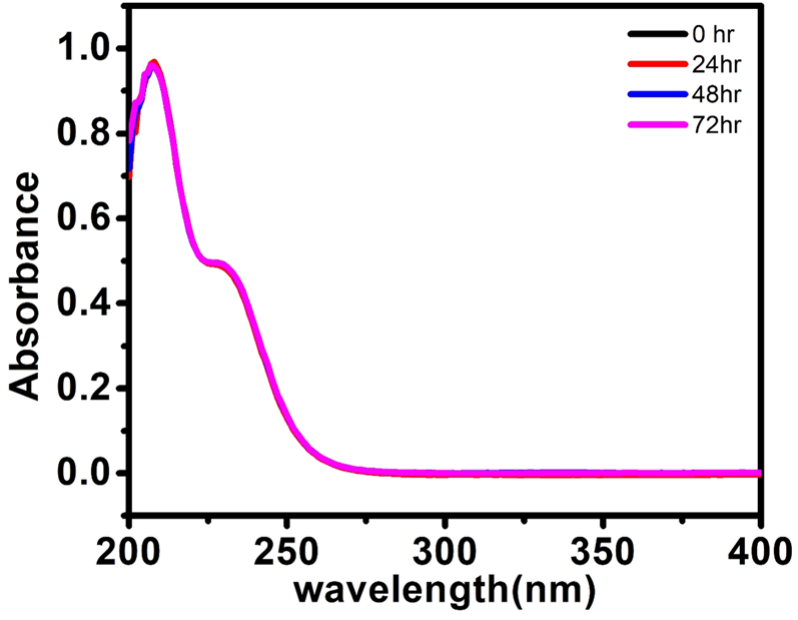
UV-visible spectra of (C_6_H_16_N)(C_6_H_15_N)_2_[Mo_8_O_26_]·3H_2_O, 1, (20 μM) in PBS buffer at 25 °C at different time intervals.

If any compound is intended for biomedical use it must be chemically stable at physiological parameters, such as 37 °C in PBS buffer (pH 7.4). The stability study was done by using a UV-visible spectrophotometer for 72 h at various time intervals for (C_6_H_16_N)(C_6_H_15_N)_2_[Mo_8_O_26_]·3H_2_O, 1. The UV-visible spectrum exhibits an intense absorption band at 207 nm attributed to the π–π* charge transfer transitions of the O_t_–Mo^60^ bond (where O_t_ is a terminal oxygen) and another band at 230 nm assigned to the charge transfer transition of O_d_ → Mo^52^ (where O_d_ is bridging oxygen). The solution of the synthesized solid 1 in PBS buffer was monitored for 72 h during which no peak disappearance or shift or appearance of a new peak was observed as shown in Fig. 5 indicating that the structural integrity was maintained for a long time and hence, we may state that the synthesized material is stable under physiological conditions.

### *In vitro* anti-tumour study

When POMs are altered by organic groups, the toxic side effects can be reduced to some extent. POM-based inorganic–organic hybrids have better cell penetration, increased selectivity, and decreased toxicity because of changes in their surface structure, charge, and polarity.^61^

**Fig. 6:**
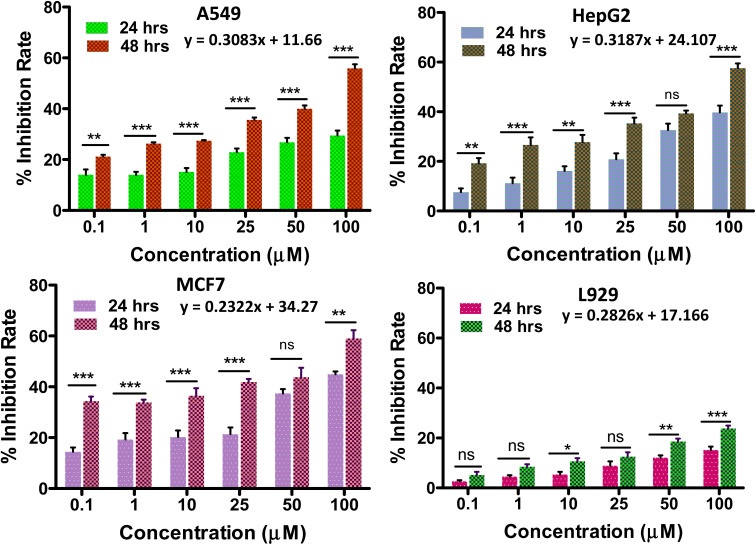
Cytotoxicity assessment(*in vitro*) of the (C_6_H_16_N)(C_6_H_15_N)_2_[Mo_8_O_26_]·3H_2_O, 1, on (A) A549, (B) HepG2, and (C) MCF-7, and (D) *in vitro* biocompatibility assay of the synthesized solid 1 on L929 cells. Experiments were performed in triplicate. And IC50 values were calculated by extrapolating the plot till 50% of the cell inhibition rate. Here, ns represents non-significant data; *P < 0.05; **P < 0.01; ***P < 0.001 (two-way ANOVA, Sidak's multiple comparisons test).

Any anti-tumor drug should be used with caution because of its potential cytotoxicity to healthy cell equivalents. Thus, the *in vitro* biocompatibility analysis of the synthesized solid 1 was performed by the conventional MTT assay on L929 fibroblast cells at varying concentrations (Fig. 6d). The biocompatibility of solid 1 was validated by the cell viability being less than 30% up to a concentration of 100 μmol L^−1^ (ref. 6 and 62) for a period of 48 hours, showing its potential to be evaluated for anti-cancer applications. In the current investigation, the impact of the POMo's incubation time on tumour growth inhibition rate was assessed (Fig. 6).

The growth inhibition rate of the cancer cells (A549, HepG2 and MCF-7) was determined by the MTT assay after treatment with different compound concentrations (0–100 μmol L^−1^) (Fig. 6) to evaluate the anti-tumour activity of the synthesized material. It was also observed that when the incubation period was increased from 24 h to 48 h the cytotoxicity potential of solid 1 was enhanced. We have further evaluated the cytotoxicity of solid 1 up to 96 hours (Fig. S9†) which provides a more comprehensive assessment of the compound's antitumor activity. The IC50 values (*i.e.* the dose required to inhibit growth of 50% of the treated cells) of solid 1 for all three cancer cell lines tested for 24 hours are found to be 57.3 μmol L^−1^, 56.2 μmol L^−1^, and 55.2 μmol L^−1^ against HepG2(Liver cancer), A549(Lung cancer), and MCF-7(Breast cancer) respectively. The above values have been compared to literature-reported IC50 values of cisplatin across all tested cell lines for 24 hours^63–65^ (Table S2†). It has been found that solid 1 consistently yielded IC50 values that are notably superior to those of cisplatin, particularly in the case of HepG2 cell lines. IC50 values can be either similar^66^ or vary^11^ among different cancer cell lines due to several factors, including variations in genetic mutations, expression of drug transporters, and sensitivity to specific compounds. These differences reflect the heterogeneity of cancer, where distinct molecular profiles and mechanisms can influence how cells respond to treatments. Understanding these variations is crucial for tailoring therapies to individual cancer types and developing more effective treatments.

### DNA fragmentation assay

DNA fragmentation assay was used to examine the pro-apoptic characteristics of solid 1 by using plasmid DNA which yielded equivalent results to genomic DNA.^67–69^ Thus, in this study, the DNA damaging potential of solid 1 was established using plasmid DNA incubated with the compound (50 μM) for 1, 2 and 3 h. After incubation, the fragmentation and smearing of the DNA were visualized upon performing gel electrophoresis. The details of the materials and methods used have been added in the ESI.† The results indicate that in comparison to the lanes 3, 4 and 5 treated with solid 1, the DNA band was more intense in the control lane. This result indicates that the cells are undergoing apoptosis as a result of treatment with the synthesized solid 1 since DNA fragmentation denotes a later stage of apoptosis showing that solid 1 causes DNA damage in a time-dependent manner. The figure has been added to the ESI† (Fig. S10).

### DNA binding studies

**Fig. 7:**
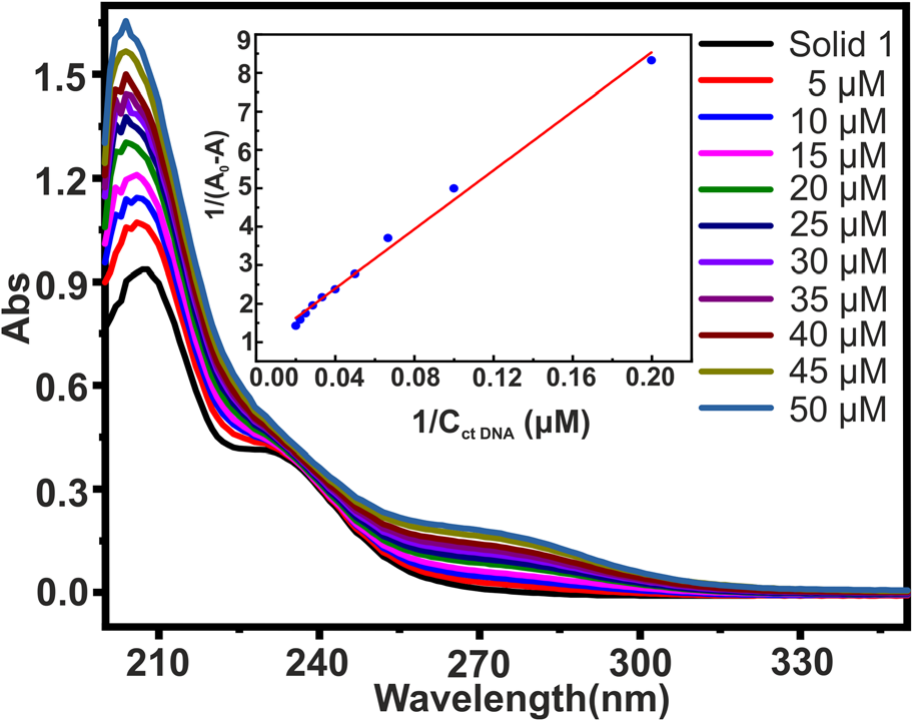
UV-visible spectroscopic study of interaction of solid 1 with ct DNA. UV-visible absorption spectra of solid 1 (20 μM) in PBS buffer (pH 7.4) with ctDNA (0–50 μM) at varying concentrations. Inset shows the double reciprocal plot of 1/(*A*_0_ − *A*) *versus* 1/*C*_ctDNA_. It was found to be linear with binding constant *K* = 2.246 × 10^3^.

The most common and effective method for evaluating the structural changes in bio macromolecules in the presence of small molecules^70^ is UV-visible absorption spectroscopy. Recently, polyoxometalates have attracted interest as agents that directly interact with DNA.^48^ The interaction of small molecules with DNA is evident from changes in the magnitude of absorbance and peak position shift which finds further correlation with the strength of the interaction.^51^ There are three main ways in which small molecules can bind to the DNA double helix:^71^ (i) intercalative binding, in which the probe is intercalated between the nucleic acid base pairs; (ii) groove binding, in which van der Waal's interaction is found in either the deep major groove or the shallow minor groove; and (iii) electrostatic interaction between the negatively charged DNA phosphate backbone and the molecules' cationic ends. To investigate the interaction between solid 1 (20 μM) and ctDNA, the UV-visible absorption spectra of 1 were measured in the absence and then in the presence of increasing concentrations of ctDNA varying from 0 to 50 μM concentration as shown in Fig. 7. The absorption spectra displayed large hyperchromic shifts (*i.e.*, increase in the absorbance with respect to the increase in the ctDNA concentration). The spectroscopic change suggests that there are interactions between synthesized solid 1 and ctDNA. This interaction can be either covalent or non-covalent. Since covalent interaction is characterized by hypochromic and bathochromic shifts, this intercalative binding is ruled out in this case. The interaction might be non-covalent or groove binding where minor/major electrostatic interaction might have caused an increase in absorption intensity.^72,73^ The double reciprocal equation (eqn. (1))^74^ expresses the absorption relationship between solid 1 and ctDNA concentration

1 1/*A*_0_ − *A* = 1/*A*_0_ + 1/ (*K* × *A*_0_ × *C*_ctDNA_)

where *A*_0_ and *A* denote the absorbances of solid 1 in the absence and presence of ctDNA at various concentrations, respectively, *K* represents the binding constant between solid 1 and ctDNA, and *C*_ctDNA_ is the concentration of ctDNA. The plot between 1/(*A*_0_ − *A*) and the reciprocal value of the ctDNA concentration (1/*C*_ctDNA_) is linear, having a slope 1/*KA*_0_. The inset in Fig. 7 evidently shows the linearity of the double reciprocal plot of 1/(*A*_0_ − *A*) *versus* 1/*C*_ctDNA_. And the value of the *K* was obtained 2.246 ×10^3^ L mol^−1^, by using eqn (1). Previously reports were published on the binding constant of well-known intercalators which are of the order 10^4^–10^6^ L mol^−1^.^75^

Although measuring the value of *K* and hyperchromism effect is not the most reliable way to find out the mode of interaction, still the fact that the value of *K* is lower than those of intercalators gives an idea that the binding mode might be non-intercalative. Hence, it is possible to hypothesise that the synthesized solid 1 binds reversibly with double stranded DNA without intercalating between DNA base pairs, and binding of 1 to the minor groove of the DNA double helix seems to be the most likely mechanism for the anti-tumour action of the synthesized solid 1. However, the hunt to deduce the exact mechanism for anti-tumor action of polyoxomolybdates is still under evaluation.

## Conclusions

To summarise, a polyoxomolybdate based organic–inorganic hybrid solid (C_6_H_16_N)(C_6_H_15_N)_2_[Mo_8_O_26_]·3H_2_O (1) was synthesized by using a hydrothermal method. POM hybrids with complementary bio(organic) or inorganic counterparts are crucial to POM engineering which can lead to domain specific superior properties. The structural elucidation revealed a nano-capsule like structure in the 50–200 nm range. Hydrogen bonding interactions are the building blocks holding the octamolybdate cluster and triethylamine ligands. The *in vitro* cytotoxicity assessment revealed this hybrid solid to be an effective anti-tumor agent against three cancer cell lines namely MCF-7 (breast cancer), A549 (lung cancer) and Hep-G2 (liver cancer) with less cytotoxicity towards normal cell lines. Further, this material was stable at physiological pH as evident from UV-vis spectroscopy making it a suitable candidate for biological applications. We have also made an attempt to understand the mechanistic pathway of anti-tumor effect followed by any {Mo_8_O_26_}^4−^ type cluster-based hybrid solid. Solid 1 was found to interact with calf-thymus DNA leaning to the fact that DNA binding can be said to be the most likely mechanism for cancer cell death. In addition to that, DNA fragmentation assays were carried out to verify the cell death by the DNA binding mechanism and revealed that the DNA was damaged upon addition of solid 1, further demonstrating that DNA binding is the most probable mechanism for the anti-tumor activity displayed by polyoxometalate-based inorganic–organic hybrid solid 1. This paves the path for exploring octamolybdate based hybrid POMs with bioorganic ligands as potent anti-tumor agents.

## Conflicts of interest

There are no conflicts to declare.

## Supplementary Material

NA-005-D3NA00459G-s001

NA-005-D3NA00459G-s002
