# Supplementary material for: A polyoxomolybdate-based hybrid nano capsule as an antineoplastic agent
Source: Nanoscale Adv. 2023 Oct 6;5(22):6045–52. doi: 10.1039/d3na00459g (PMC10628982; doi:10.1039/d3na00459g)
Supplement: NA-005-D3NA00459G-s001 [file NA-005-D3NA00459G-s001.pdf]

Electronic Supplementary Material (ESI) for Nanoscale Advances.
This journal is © The Royal Society of Chemistry 2023

Polyoxomolybdate-based hybrid nano capsule as an antineoplastic agent

Arti Joshi, [†a] Sobhna Acharya,[†a] Neeta Devi, [a] Ruby Gupta, [a] Deepika Sharma[a] and Monika Singh* [a]

[a] Institute of Nano Science and Technology, Knowledge city, Sector-81, Mohali, Punjab, INDIA
† Both the authors have equal contribution

Electronic Supplementary Information (ESI)

Single Crystal X-Ray Diffraction. To study single crystal X-ray diffraction, a crystal of appropriate size was mounted on a capillary. Using a BRUKER AXS SMART-APEX three-circle diffractometer with a CCD area detector (K α = 0.71073 Å, monochromator: graphite) data was collected.[1]Frames were obtained at T = 293 or 100 K by ω, ϕ and 2θ-rotation at 10 s per frame with **SAINT**.[2] The measured intensities were reduced to F^2 and was corrected for absorption with **SADABS**. **SHELXTL**[3] was used to solve the structure, refinement, and data output. Diamond[4] program was used to create the images. The summary of crystallographic data is provided in table S1.

Other Analytical Techniques. Using Cu-K radiation (1.5418), Powder X-Ray Diffraction (PXRD) investigations were carried out on a Bruker D8 − Advanced Eco X-ray Diffractometer. Agilent Cary 600 series Fourier transform infrared (FT-IR) spectrometer was used to do FT-IR spectroscopic measurements (with KBr) in the 400–4000 cm^{-1} range. Perkin Elmer's STA-8000 performed the thermal analysis (TGA) on well-ground samples in a flowing nitrogen atmosphere at a heating rate of 10 ◦C/min. The morphology of the solid **1** was investigated by scanning electron microscope (SEM) from JEOL (JSM IT-300) being provided with an energy-dispersive X-ray diffractometer(Bruker) and transmission electron microscopy (TEM); a JEM-2100 instrument which is equipped with an energy-dispersive X-ray spectrometer, operated at an accelerated voltage of 200 kV. The NEXSA X-Ray Photoelectron Spectrometer (XPS) instrument of Thermo scientific was used to determine the valence states of the elements.

In Vitro Biocompatibility and Cytotoxicity Assay: For in-vitro toxicity measurements, a 100 μM stock solution of the solid 1 was prepared in PBS Buffer. The solid was observed to be completely soluble in the solution. The biocompatibility of the solid1 was determined on the L929 cell line, while the cytotoxic effect of the solid 1 was evaluated on A549, MCF-7, and HepG2 cancer cells by the conventional MTT assay.37 Briefly, 100 μL of the cell suspension containing 1 × 10^4 cells/mL was seeded in a 96-well culture plate. After 24 h of incubation at 37 °C under 5% CO_2, the cells were then treated with an aqueous solution of solid 1 at varied concentrations (0.1−100 μM) for 24, 48,72 and 96 hours. After the appropriate treatment period, 10 μL of MTT [3-(4, 5-dimetheylthiazol-2)-2,diphenyl tetrazolium bromide] reagent solution in PBS (5 mg/mL) was added and then incubated for an additional 4 h at 37 °C. Fifty microliters of DMSO was then added to solubilize the formazan crystals formed, and the optical density was determined at 595 nm using a BIO-

RAD microplate reader. The relative tumor inhibition rate (%) was then determined using the equation:

$$\text{inhibition rate (\%)} = [100 - A_t/A_c] * 100$$

where At represents absorbance of treated cells and Ac represents the absorbance of untreated control cells. For each of the cell lines used,all the experiments were performed in triplicate. GraphPad Prism 6 was used to determine the statistical significance of the data obtained.
If a P value less than 0.05 was achieved, the data was declared as statistically significant as examined by two-way ANOVA analysis keeping a 95% confidence interval.

DNA Fragmentation assay: To check the DNA damage caused by Solid 1 DNA Fragmentation assay was done. Here, we use the 100ng of plasmid(pEGEP) DNA which is incubated with concentration of (50µM) Solid 1 in DNAase free water at hourly time intervals for 3 hours.The reactions were then resolved on a 1.5% agarose gel and photographed.

Table S1. Crystal data and structural refinements for **solid 1**

**Parameter**	**$(C_6H_{16}N)(C_6H_{15}N)_2[Mo_8O_{26}].3H_2O$**
Formula	C18 H52 Mo8 N3 O29
Formula weight, g	1542.14 g/mol
T (K)	293(2)
Wavelength (Å)	0.71073
Crystal system	Monoclinic
Space Group	P 1 21/c 1 (14)
a (Å)	20.202(4) Å
b (Å)	11.834(2) Å
c (Å)	21.348(4) Å
α (°)	90.00
β (°)	118.24(3)
γ (°)	90.00
V ($Å^3$)	4496.20(7246) $Å^3$
Z	4
dcalc (gcm^{-3})	2.27804 g/cm^3
μMoKα, (cm^{-1})	7.334
R1(I>2σI)	0.0419
WR2(all)	0.1111
CCDC/CSD No.	2189304

Table S2. IC50 values of Solid 1 and cisplatin in Hep-G2, A549, MCF-7

Cell line	Solid 1	Cisplatin
HepG2	46	66.13
A549	39	24.4
MCF-7	18	5.75

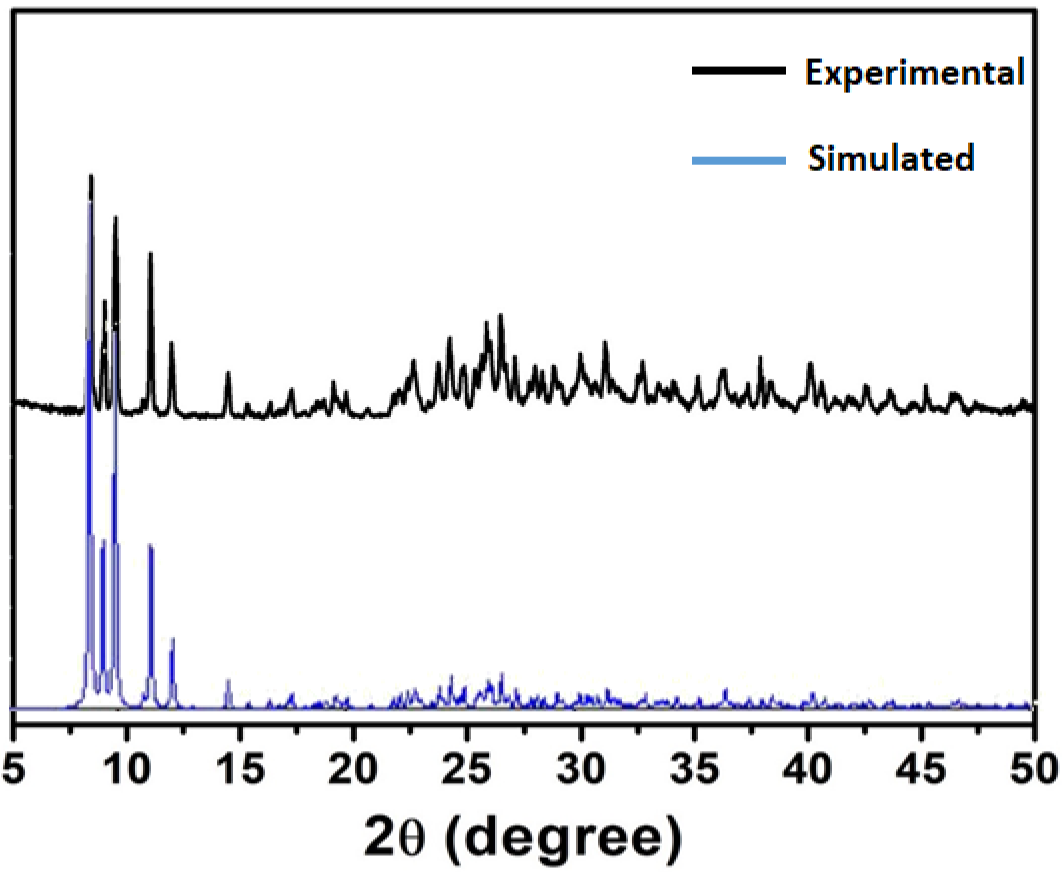

Fig S1. Simulated (blue) and experimental (black) XRD patterns of $(C_6H_{16}N)(C_6H_{15}N)_2[Mo_8O_{26}].3H_2O$ (**1**)

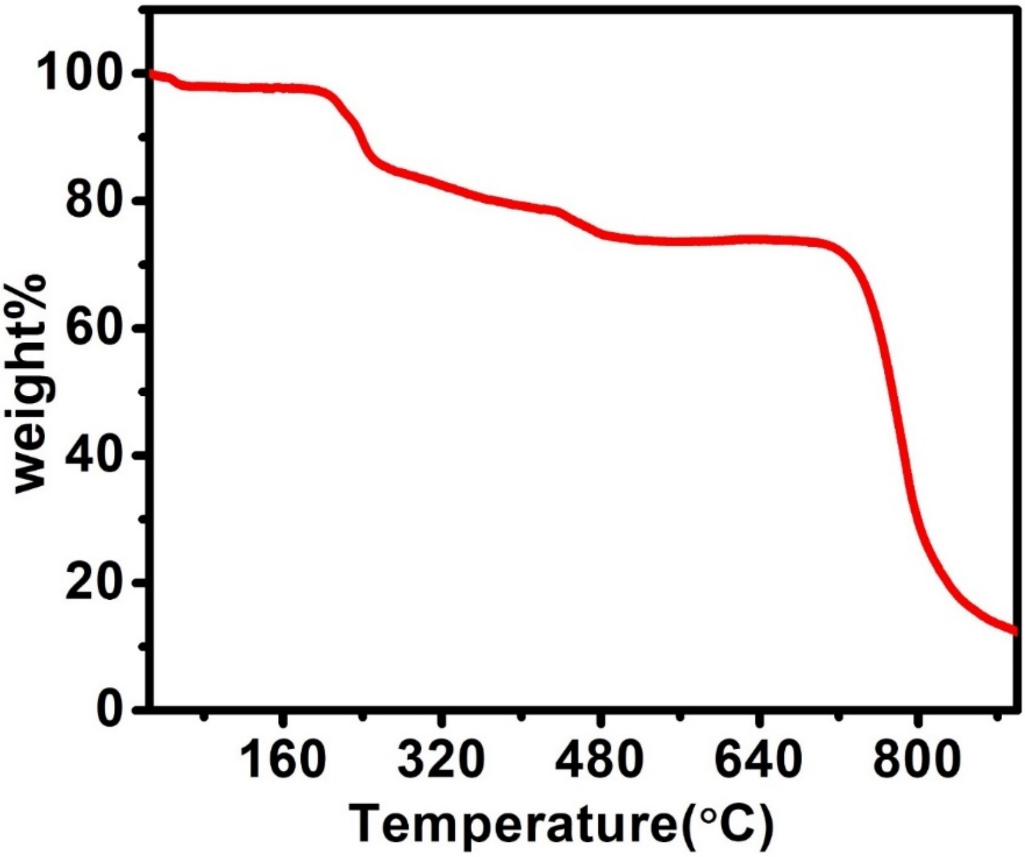

Fig S2. Thermogravimetric analysis curve of $(C_6H_{16}N)(C_6H_{15}N)_2[Mo_8O_{26}].3H_2O$,solid **1**

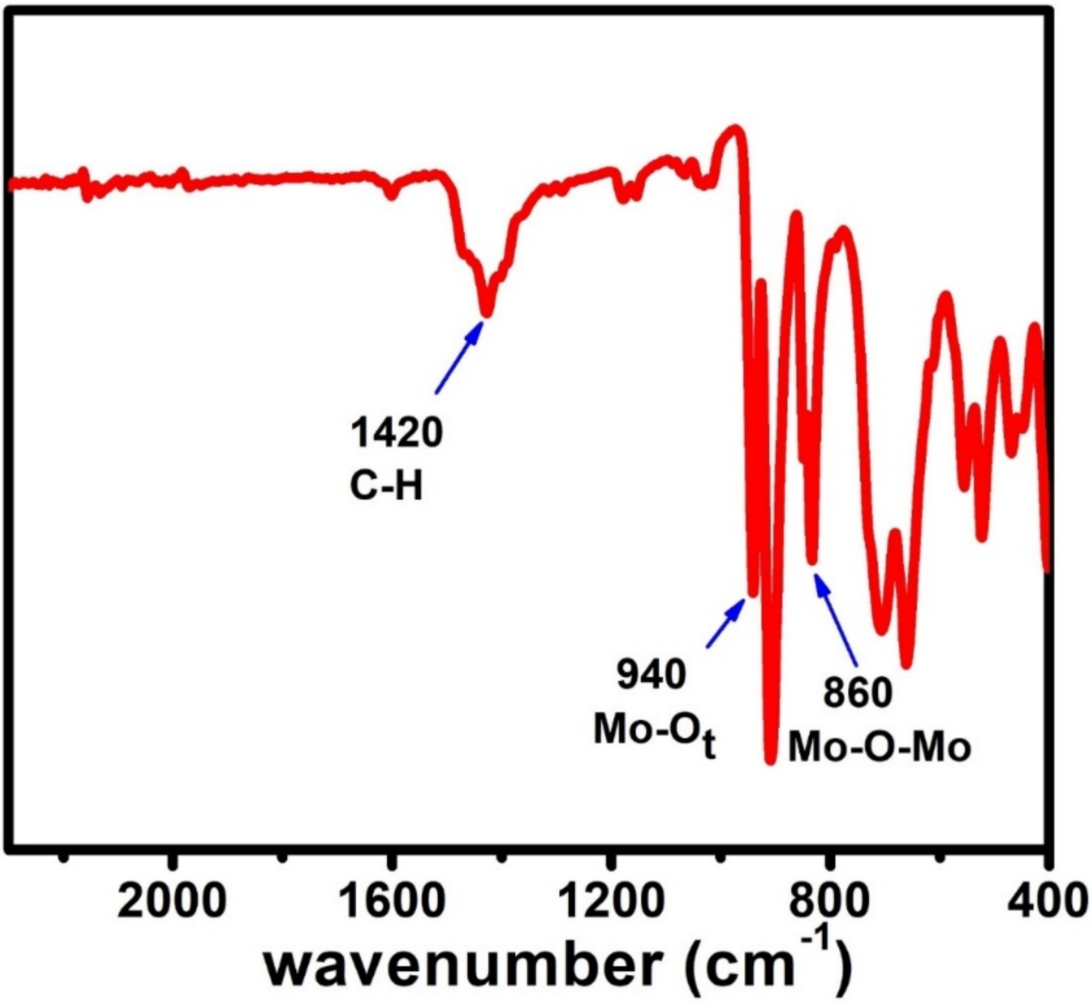

Fig S3. FTIR curve of $(C_6H_{16}N)(C_6H_{15}N)_2[Mo_8O_{26}].3H_2O$ (**1**)

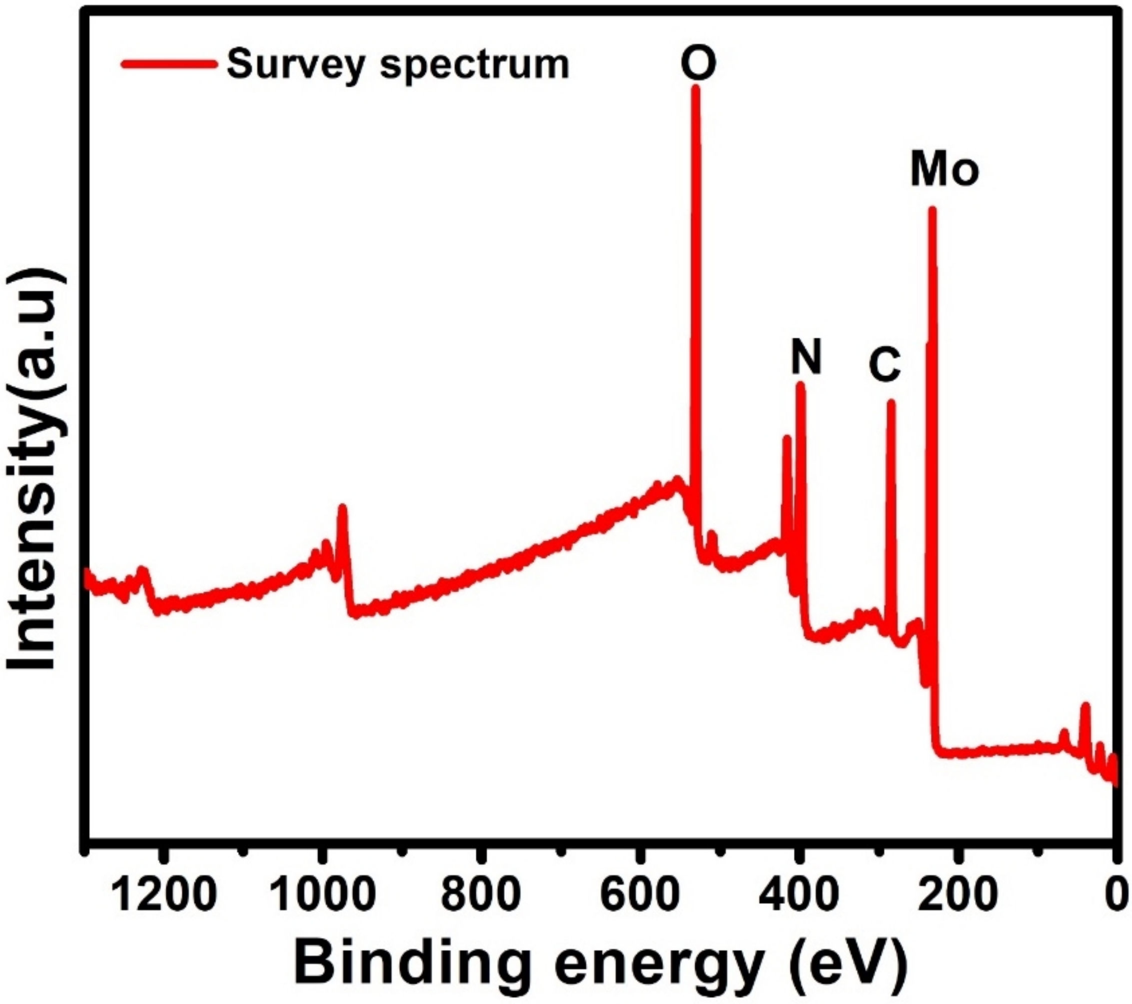

Fig S4. XPS Survey spectrum of $(C_6H_{16}N)(C_6H_{15}N)_2[Mo_8O_{26}].3H_2O$ (**1**)

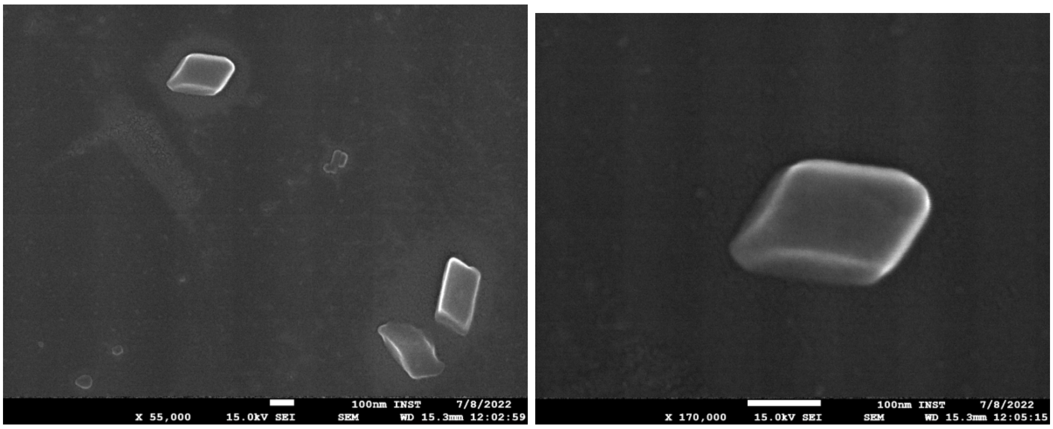

Fig S5. FESEM images of $(C_6H_{16}N)(C_6H_{15}N)_2[Mo_8O_{26}].3H_2O$ (**1**)

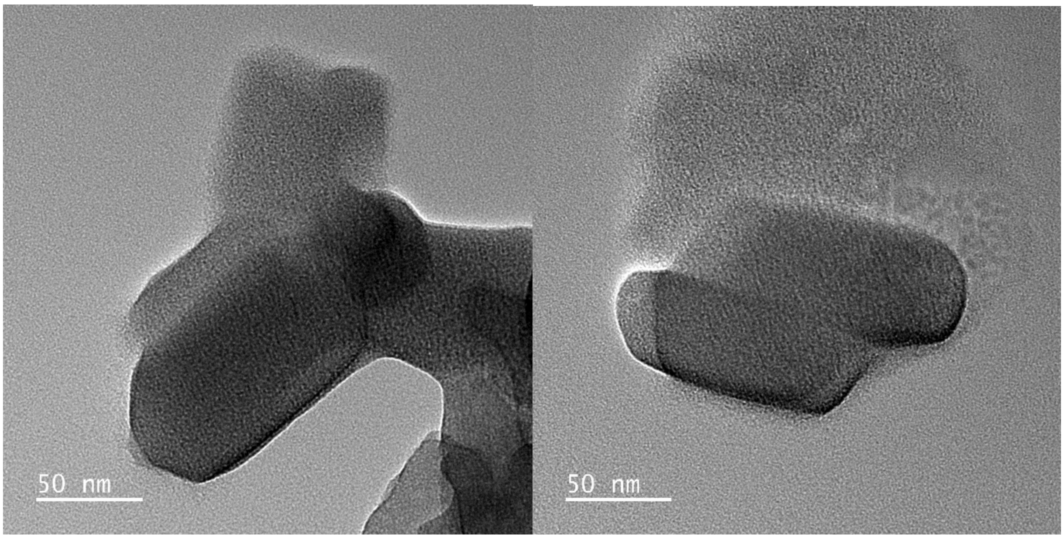

Fig S6. TEM images of $(C_6H_{16}N)(C_6H_{15}N)_2[Mo_8O_{26}].3H_2O$ (**1**)

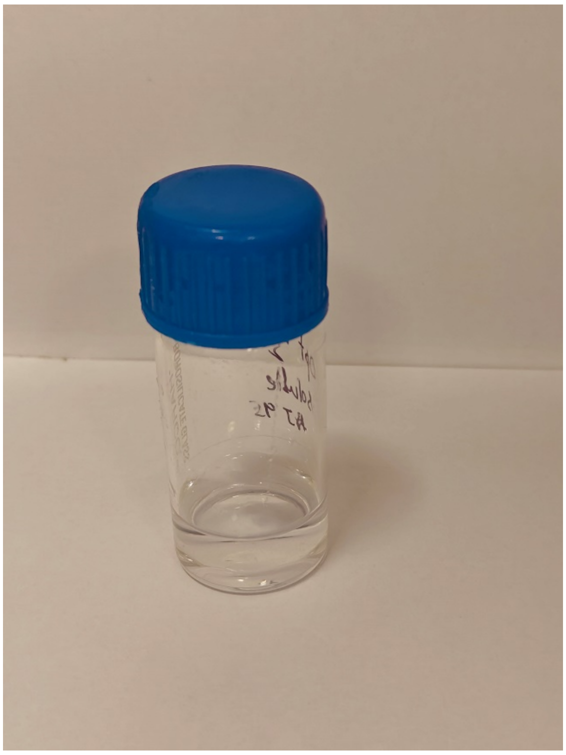

Fig S7. Solubility of solid1 in water(1.8mg/ml)

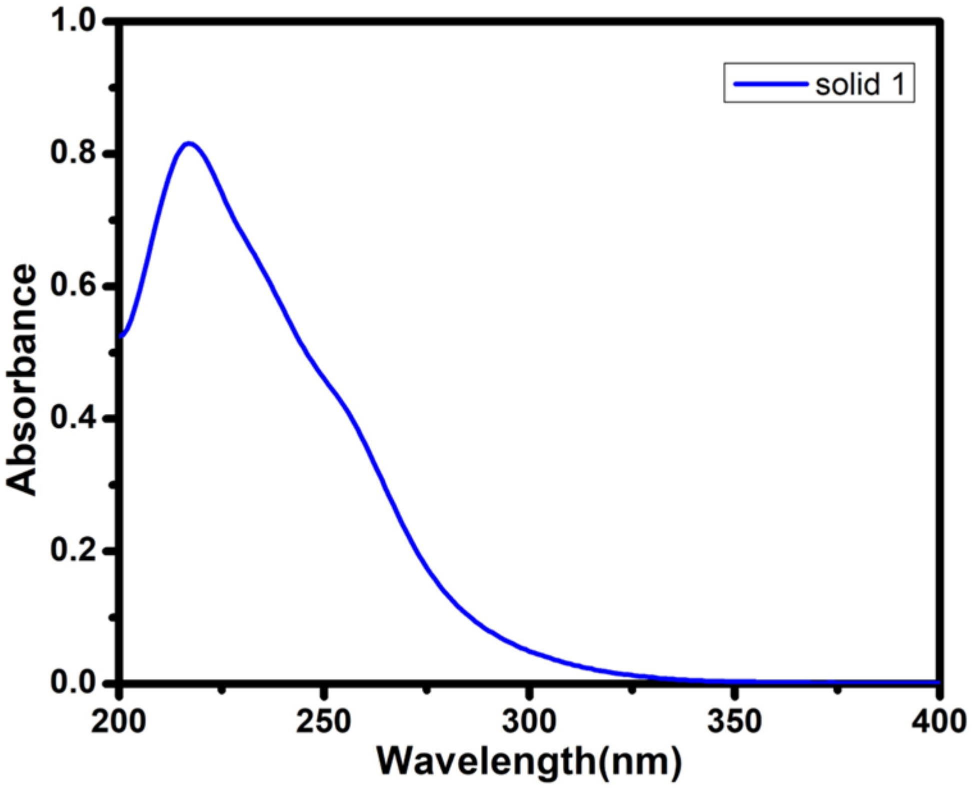

Fig S8. Stability of solid1 in water after dispersing it for 72hrs

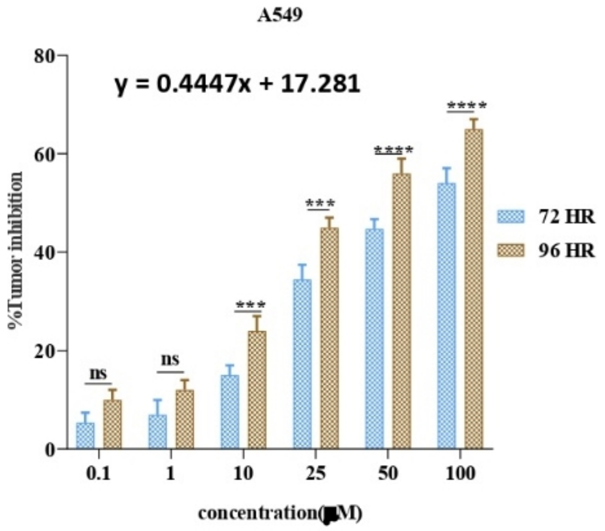

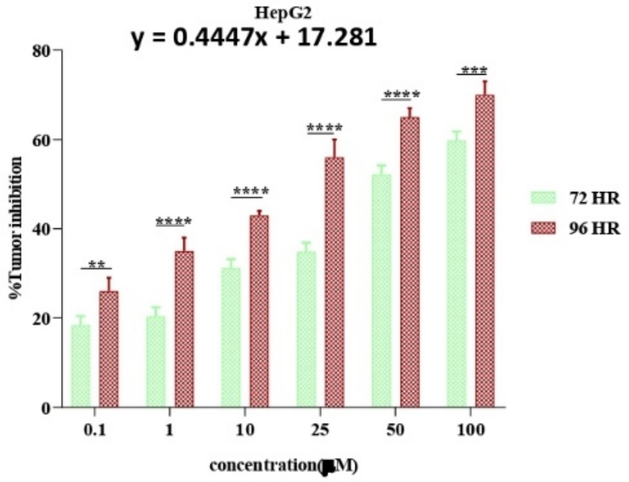

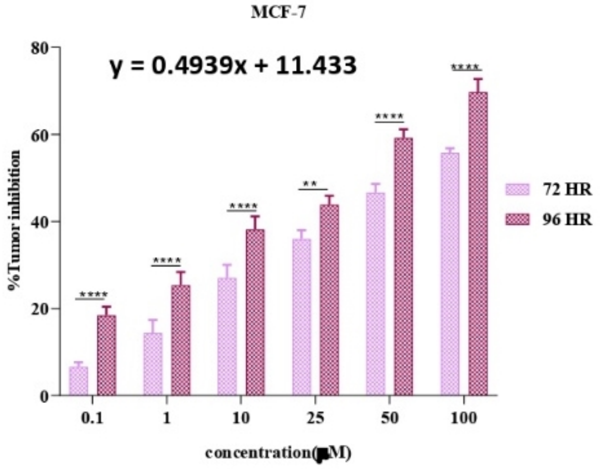

Cell line	IC50
A549(Lung Cancer)	29.45(µM)
HepG2 (Liver Cancer)	34.87(µM)
MCF(Breast Cancer)	19.08(µM)

Fig. S9. Cytotoxicity evaluation of the $(C_6H_{16}N)(C_6H_{15}N)_2[Mo_8O_{26}].3H_2O$, 1 on various cancer cell lines: (A) HepG2, (B) A549, (C) MCF-7

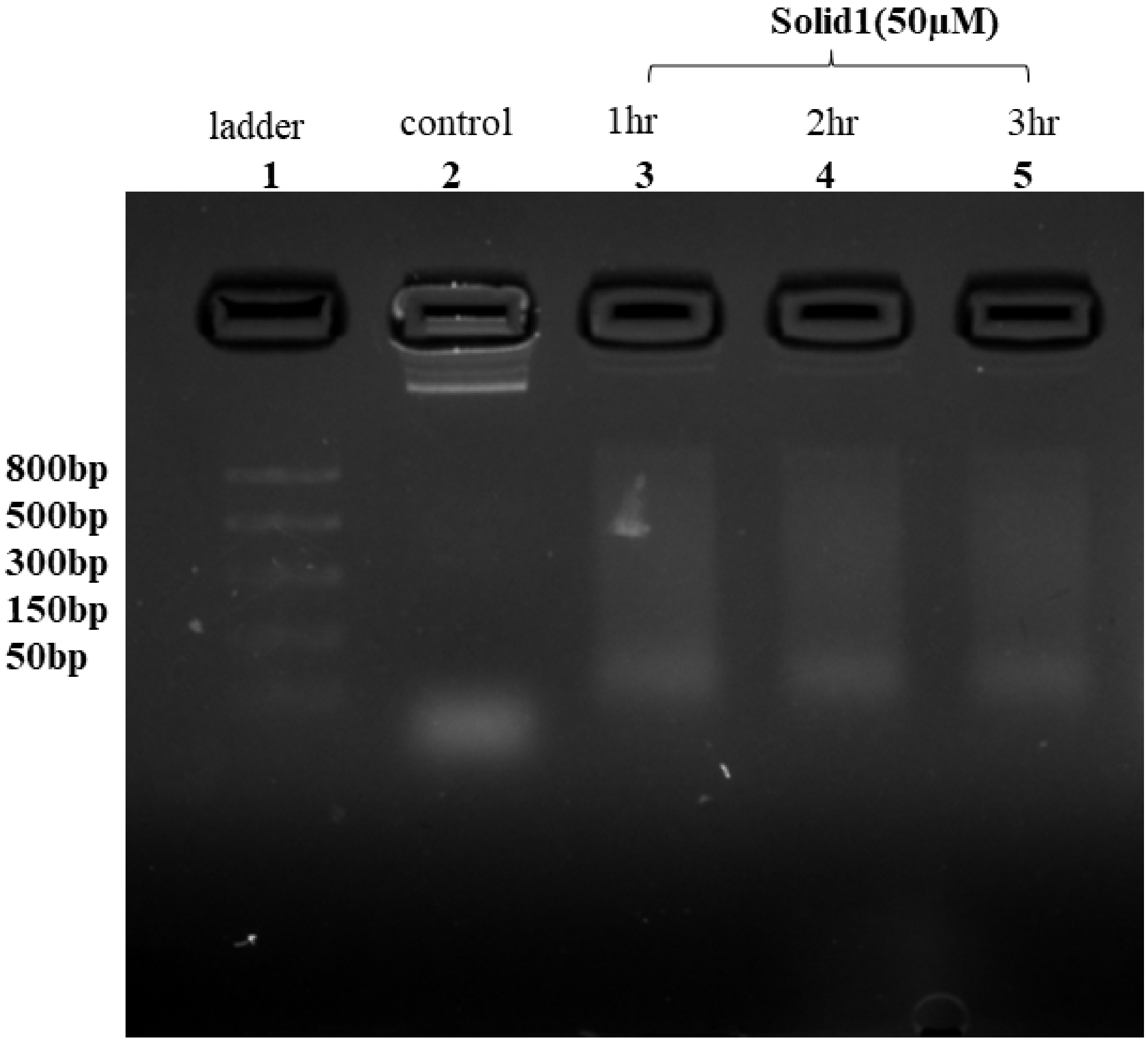

Fig S10. DNA fragmentation assay demonstrates DNA Damage, Supporting Anti-Tumor Mechanisms

References:

(1) Bruker Analytical X-ray Systems, SMART: Bruker Molecular Analysis Research Tool, Version 5.618; Bruker AXS: Madison, WI, 2000.

(2) Bruker Analytical X-ray Systems, SAINT-NT, Version 6.04; Bruker AXS: Madison, WI, 2001.

(3) Bruker Analytical X-ray Systems, SHELXTL-NT, Version 6.10; Bruker AXS: Madison WI 2000. S12

(4) Klaus, B. DIAMOND, version 1.2c; University of Bonn: Germany, 1999
